# SUMOylation as a Stress-Responsive Rheostat in Cardiovascular Disease

**DOI:** 10.3390/cells15201838

**Published:** 2026-10-10

**Authors:** Qiaoling Su, Yuejie Sun, Tongyao Zhuang, Qi Cheng, Wei Peng

**Affiliations:** 1 Key Laboratory of Evolution & Marine Biodiversity (Ministry of Education) and Institute of Evolution & Marine Biodiversity, Ocean University of China, Qingdao 266003, China; suqiaoling@stu.ouc.edu.cn; 2 Fisheries College, Ocean University of China, Qingdao 266003, China; 3 Haide College, Ocean University of China, Qingdao 266100, China; 4 Xuzhou Central Hospital, Southeast University, Xuzhou 221009, China; 5 Institute of Organ Transplantation, Tongji Hospital, Tongji Medical College, Huazhong University of Science and Technology, Wuhan 430000, China

**Keywords:** SUMOylation, oxidative stress, inflammation, cardiovascular disease, mitochondria, cardiac remodeling, ischemia–reperfusion injury

## Abstract

SUMOylation is a reversible post-translational modification that regulates protein stability, localization, activity, and protein–protein interactions. In the cardiovascular system, SUMO signaling intersects with oxidative stress, mitochondrial homeostasis, calcium handling, inflammatory activation, and stress-induced transcriptional reprogramming. Here, we review the molecular architecture of the SUMO system and its redox sensitivity and integrate evidence for SUMO-dependent regulation of mitochondrial quality control, metabolic adaptation, inflammatory responses, and regulated cell death. We further examine how these mechanisms contribute to atherosclerosis, myocardial ischemia–reperfusion injury, cardiac remodeling, heart failure, arrhythmia, and pulmonary vascular disease, while distinguishing evidence that is mechanistic, correlative, or contradictory. Based on this evidence, we propose a stress-responsive SUMO rheostat framework in which the functional output of SUMO signaling is shaped not simply by the magnitude of stress, but by the interplay among stress intensity and duration, subcellular compartment, substrate accessibility and abundance, SUMO paralogue composition, enzyme activity, cell identity, and disease stage. These contextual variables can influence which SUMO-regulated substrates are accessible to individual enzymes, where modification occurs, and how long it persists, thereby contributing to divergent functional outcomes under otherwise similar stress conditions. The framework is intended as a conceptual synthesis for reconciling seemingly divergent effects of SUMOylation across cardiovascular diseases rather than as a quantitatively validated stress–SUMO response model. Finally, we discuss the translational challenges and opportunities of moving beyond global modulation of the SUMO pathway toward substrate-selective, enzyme-selective, and cell-targeted therapeutic strategies.

## 1. Introduction

### 1.1. Oxidative–Inflammatory Imbalance in Cardiovascular Disease

Cardiovascular diseases (CVDs) comprise a heterogeneous group of disorders driven by the interaction of metabolic, mechanical, oxidative, inflammatory, and cell-autonomous stress signals. According to the latest Global Burden of Disease (GBD) 2023 estimates, CVDs accounted for approximately 437 million disability-adjusted life years (DALYs) and 19.2 million deaths worldwide in 2023, representing 15.6% of all-age DALYs. Ischemic heart disease (IHD), a major clinical manifestation of atherosclerotic cardiovascular disease, accounted for approximately 193 million DALYs, while hypertensive heart disease contributed 28.4 million DALYs globally. In addition, approximately 223 million CVD DALYs were attributable to high systolic blood pressure in 2023, highlighting the substantial global burden associated with elevated blood pressure [1].

Although atherosclerosis, arterial hypertension, cardiac hypertrophy, heart failure, and myocardial ischemia–reperfusion (I/R) injury differ in their initiating stimuli and temporal trajectories, they share recurrent pathogenic features, including endothelial dysfunction, immune-cell recruitment, mitochondrial injury, and maladaptive tissue remodeling [2,3,4]. Atherosclerosis is characterized by lipid accumulation, chronic vascular inflammation, and intimal plaque formation, whereas myocardial I/R injury is dominated by acute metabolic collapse, oxidative stress, calcium overload, and sterile inflammation [4,5]. Hypertension is associated with endothelial dysfunction, immune activation, oxidative stress, and vascular remodeling; in the myocardium, sustained pressure overload initially induces compensatory hypertrophy but ultimately promotes maladaptive remodeling and heart failure [3,6,7,8,9,10].

Reactive oxygen species (ROS) should not be regarded solely as toxic by-products of metabolism. At physiological levels, ROS participate in redox signaling and coordinate transcription, metabolism, and stress adaptation. In contrast, excessive or prolonged ROS generation can overwhelm antioxidant defenses and damage proteins, lipids, and nucleic acids [11,12,13,14,15]. The biological consequences of ROS depend on the chemical species involved, the source of production, the magnitude and duration of exposure, and the subcellular compartment in which they act [12,13,14,15]. Oxidative and inflammatory signaling are reciprocally coupled in CVD. ROS can activate MAPK-, NF-κB-, and inflammasome-associated pathways, whereas inflammatory mediators can further increase ROS production through NADPH oxidases, mitochondrial dysfunction, and impaired antioxidant systems [2,7,14,16,17]. These feed-forward circuits contribute to endothelial activation, vascular injury, fibro-inflammatory remodeling, and cardiomyocyte dysfunction across atherosclerosis, hypertension, heart failure, and I/R injury [2,3,4,7,16,17]. However, oxidative stress and inflammation are not exclusively detrimental processes; both also participate in adaptive responses that maintain cellular homeostasis and promote tissue repair. This duality represents a fundamental challenge in cardiovascular biology, as the molecular determinants that govern the transition from adaptive stress responses to maladaptive injury remain incompletely understood. Identifying regulatory systems capable of integrating stress intensity, duration, and cellular context is therefore essential for understanding disease progression and developing more precise therapeutic strategies.

Post-translational modifications provide an efficient molecular mechanism for integrating rapidly changing stress signals. Among them, SUMOylation is particularly well suited for this role because it is reversible, dynamically regulated, and capable of altering protein stability, subcellular localization, enzymatic activity, and protein–protein interactions without necessarily directing substrates toward degradation [18,19,20,21,22]. The SUMO system conjugates SUMO proteins to lysine residues through an E1–E2–E3 enzymatic cascade, whereas SUMO-specific proteases (SENPs) remove SUMO and maintain dynamic homeostasis [18,19,20,21,23,24]. In the cardiovascular system, this regulatory architecture is particularly relevant because oxidative stress, mitochondrial dysfunction, calcium dysregulation, hypoxia, and inflammatory activation frequently coexist during disease progression [21,22,23,24].

Importantly, the cardiovascular consequences of SUMOylation are substrate-specific and context-dependent. Rather than treating SUMOylation as uniformly beneficial or detrimental, it is more useful to view it as a stress-responsive regulatory layer that shapes the output of signaling networks according to substrate identity, SUMO paralogue, cell type, disease stage, and local biochemical environment [25,26,27,28]. This framework provides the basis for the disease- and mechanism-specific discussion that follows.

### 1.2. Scope and Rationale of This Review

Several reviews have summarized the role of SUMOylation in cardiovascular biology and disease, including its involvement in cardiac development, heart failure, ischemic injury, mitochondrial regulation, and the functions of SUMO-specific proteases [21,22,23,24,27]. The present review adopts a pathway-oriented strategy rather than presenting SUMOylated proteins as a long substrate catalogue.

First, we examine how the SUMO machinery responds to oxidative stress. Particular emphasis is placed on the redox sensitivity of SUMO-conjugating enzymes and SENPs, including changes in enzyme stability, localization, and substrate accessibility [25,29,30,31,32,33]. This redox responsiveness suggests that SUMO signaling is not simply downstream of oxidative stress but may itself constitute part of the cellular stress-sensing apparatus.

Second, we organize the available evidence around mitochondrial quality control, calcium handling, metabolic adaptation, inflammatory licensing, endothelial homeostasis, and vascular remodeling. These processes are interconnected and are regulated by SUMO-dependent mechanisms at multiple levels, including mitochondrial dynamics, SERCA2a function, PPARγ signaling, NLRP3 activation, ERK5 regulation, and endothelial nitric oxide production [34,35,36,37,38,39,40,41,42,43].

Building on these considerations, we propose a stress-responsive “SUMO rheostat” framework in which the functional output of SUMO signaling is shaped by stress intensity and duration, subcellular redox compartment, SUMO paralogue composition, substrate accessibility and abundance, E3-ligase and SENP activity, cell identity, and disease stage [25,26,27,28,44]. Rather than functioning as a binary switch, SUMOylation may modulate the amplitude, duration, and localization of cellular stress responses in a context-dependent manner. This framework provides a conceptual basis for integrating the molecular mechanisms, cell-specific effects, and disease-associated outcomes discussed throughout this review.

This review therefore emphasizes three principles. First, SUMO signaling should be interpreted according to stress intensity, duration, and cellular context. Second, oxidative and inflammatory SUMO signaling should be considered as bidirectionally coupled networks rather than isolated modules. Third, therapeutic development should move from global manipulation of the SUMO cascade toward substrate-selective, enzyme-selective, and cell-targeted interventions.

## 2. Overview of the SUMOylation System

SUMOylation is a reversible post-translational modification in which small ubiquitin-like modifier (SUMO) proteins are covalently attached to lysine residues of target proteins. By modifying protein activity, stability, localization, and interactions, SUMOylation enables rapid adaptation of cellular signaling networks to changing intracellular conditions [18,19,20,27]. In the cardiovascular system, this regulatory process is particularly relevant because oxidative stress, mitochondrial dysfunction, calcium dysregulation, hypoxia, and inflammatory activation frequently coexist during disease progression [21,22,23,24].

### 2.1. Core SUMOylation Machinery

SUMO1 and SUMO2/3 are the principal SUMO paralogues in mammalian cells. SUMO conjugation is mediated by an ATP-dependent enzymatic cascade involving the SUMO-activating enzyme SAE1/SAE2, the E2 conjugating enzyme UBC9, and, for selected substrates, SUMO E3 ligases. Conversely, SENPs remove SUMO from target proteins and maintain the dynamic balance between SUMOylation and deSUMOylation [18,19,20,21,23,24]. This reversible enzymatic system allows SUMOylation to respond rapidly to changes in cellular stress and signaling states.

Beyond the core enzymatic machinery, however, the functional interpretation of SUMOylation requires consideration of how these components respond to cellular stress and how their activity is reflected at the substrate level. Importantly, the biological consequences of SUMOylation cannot be inferred solely from changes in global SUMO-conjugate levels. Global SUMO-conjugate abundance reflects the net balance of SUMO conjugation and deSUMOylation across numerous substrates but does not identify the proteins responsible for the observed change or establish their functional consequences. Substrate-associated SUMOylation provides stronger evidence that a specific protein is modified, although such analyses may not resolve the relevant SUMO paralogue or acceptor residue. Site-resolved approaches, including residue-specific mutagenesis and proteomic mapping, provide more direct mechanistic evidence by linking a defined SUMO modification to changes in substrate function. However, site-level evidence remains available for only a subset of cardiovascular SUMO substrates. Accordingly, interpretation of SUMO-dependent mechanisms requires consideration of the modified substrate, SUMO paralogue, modification site, cell type, subcellular compartment, and stress context [18,19,20,21,44].

### 2.2. Redox Sensitivity Links SUMOylation to Cardiovascular Stress

The SUMOylation machinery is sensitive to oxidative stress because several components contain catalytically important or redox-sensitive cysteine residues. Oxidative conditions can alter the activity, stability, localization, or substrate accessibility of SUMO-conjugating and deconjugating enzymes, thereby coupling cellular redox status to the SUMO modification landscape [25,31,33]. SENP family members are particularly relevant because their abundance and activity can be regulated by oxidative stress [29,30,31,32]. Importantly, such changes in the SUMO machinery do not necessarily produce a uniform increase or decrease in SUMOylation across all substrates.

**Figure 1:**
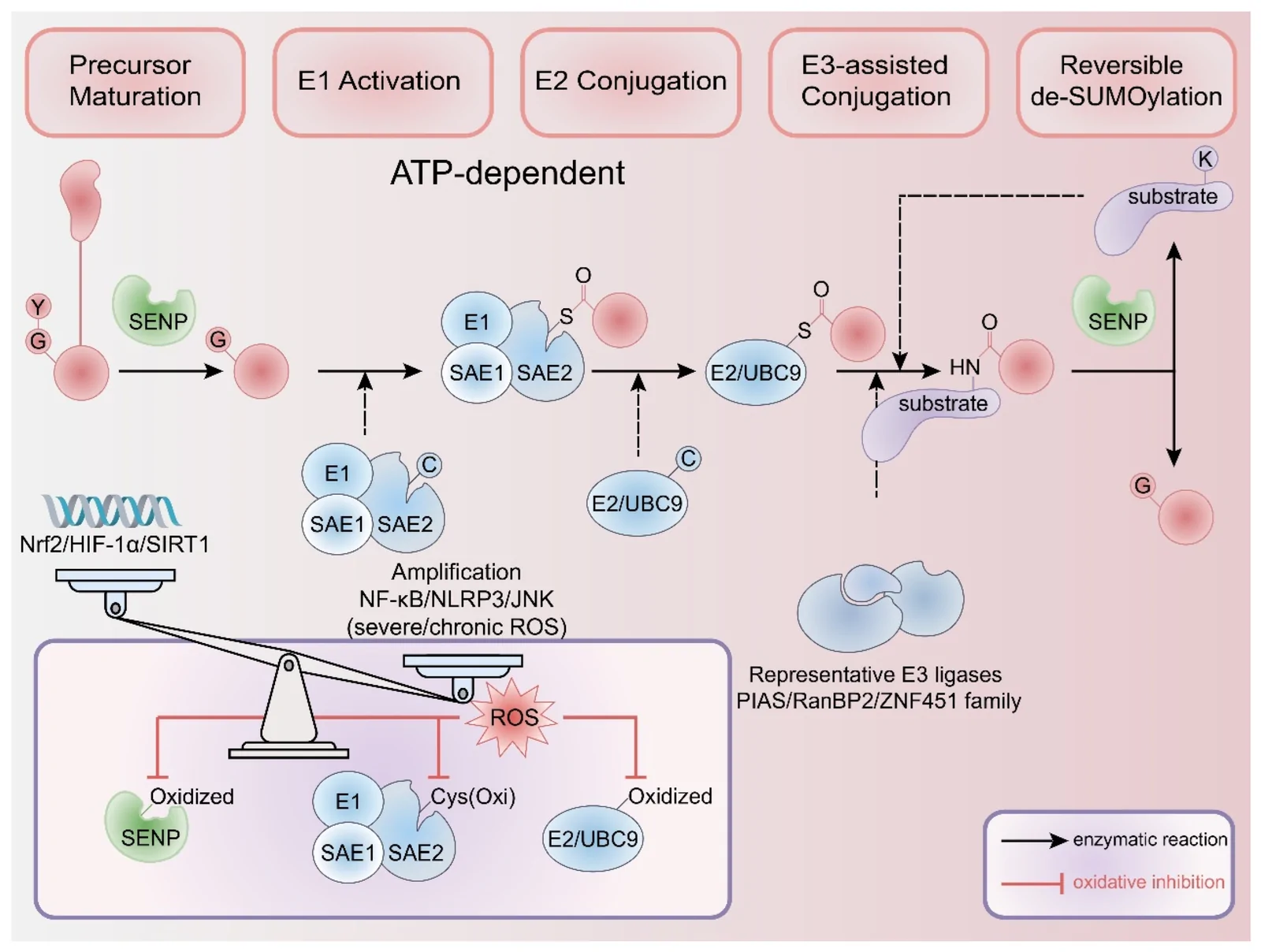
Canonical SUMOylation machinery and redox-sensitive regulation.

As summarized in Figure 1, the upper panel illustrates the canonical SUMOylation cycle, including SUMO maturation, E1-mediated activation, E2/UBC9-mediated transfer, E3-assisted substrate conjugation, and reversible de-SUMOylation by SENPs. The lower panel provides a conceptual overview of how oxidative stress may influence SUMO-regulatory capacity and downstream cellular responses. The balance-like schematic is intended to represent a conceptual relationship between oxidative stress burden and SUMO-regulatory capacity rather than a quantitatively validated stress–SUMO response curve. The figure therefore emphasizes that the functional output of SUMO signaling is shaped by the cellular redox environment and by the context-dependent regulation of the SUMO machinery.

A well-characterized example is SENP3, whose abundance and activity are regulated by oxidative stress. In non-cardiovascular models, SENP3 stabilization under mild oxidative stress promotes p300 deSUMOylation and enhances HIF-1 transcriptional activity [30,32]. SENP3 can also be regulated through interactions involving CHIP and Hsp90, providing an additional mechanism by which oxidative conditions influence SUMO protease stability [30,31,32]. Although these mechanisms provide an important framework for understanding redox-dependent regulation of SUMO signaling, much of the underlying evidence was obtained outside the cardiovascular system. Their quantitative contribution to specific cardiovascular stress responses therefore remains to be established.

More broadly, the functional consequences of altered SUMOylation are substrate- and context-dependent. Studies of individual substrates indicate that SUMO-dependent regulation may differ according to the SUMO paralogue, modification site, modifying or deconjugating enzyme, cellular compartment, and stress intensity or duration [45,46]. Thus, global changes in SUMO-conjugate abundance provide evidence of remodeling of the SUMO system but should not be used as a surrogate for the direction of regulation of an individual cardiovascular substrate [44,47]. Mechanistic inference is stronger when substrate-associated or site-resolved SUMOylation is linked to functional analysis of the modified protein [44,47,48].

The SUMOylation system is a dynamic regulatory network that integrates cellular stress signals through reversible protein modification and de-SUMOylation. The upper panel illustrates the canonical SUMOylation cycle, including precursor maturation, ATP-dependent E1 activation, E2/UBC9-mediated conjugation, E3-assisted substrate modification, and reversible de-SUMOylation by SENPs. The E1 heterodimer SAE1/SAE2 activates mature SUMO, which is transferred to E2/UBC9 and subsequently conjugated to substrate proteins with assistance from E3 ligases, including representative PIAS, RanBP2, and ZNF451 family members. SENPs mediate reversible de-SUMOylation and thereby contribute to dynamic SUMO cycling.

The lower panel illustrates how oxidative stress may remodel the SUMO machinery and downstream signaling. Moderate or transient oxidative stress may alter the stability, localization, and activity of redox-sensitive SUMO regulators, including SENP3, and influence stress-adaptive pathways such as Nrf2, HIF-1α, and SIRT1-associated signaling. Under severe or chronic oxidative stress, increased ROS may impair components of the SUMO-conjugation machinery and alter SUMO-dependent signaling, potentially contributing to NF-κB-, NLRP3-, and JNK-associated inflammatory responses. The balance-like schematic represents a conceptual relationship between oxidative stress burden and SUMO-regulatory capacity rather than a quantitative stress–SUMO response curve. The figure therefore emphasizes that oxidative stress may alter SUMO-regulatory capacity in a context-dependent manner, with the resulting effects depending on the state and activity of the SUMO machinery.

Abbreviations: SUMO, small ubiquitin-like modifier; SENP, sentrin/SUMO-specific protease; E1, SUMO-activating enzyme; SAE1/2, SUMO-activating enzyme subunit 1/2; E2, SUMO-conjugating enzyme; UBC9, ubiquitin-conjugating enzyme 9; E3, SUMO E3 ligase; PIAS, protein inhibitor of activated STAT; RanBP2, Ran-binding protein 2; ZNF451, zinc finger protein 451; Nrf2, nuclear factor erythroid 2-related factor 2; HIF-1α, hypoxia-inducible factor 1-alpha; SIRT1, sirtuin 1; NF-κB, nuclear factor kappa B; NLRP3, NLR family pyrin domain containing 3; JNK, c-Jun N-terminal kinase; ROS, reactive oxygen species; ATP, adenosine triphosphate; Cys-Oxi, oxidized cysteine residues; Y, tyrosine; G, glycine; C, cysteine; K, lysine; OX, oxidative inhibition/modification. Black arrows indicate enzymatic reactions, dashed arrows indicate the recruitment or participation of the indicated components in the corresponding steps, and red blunt-ended lines indicate oxidative inhibition.

### 2.3. SUMOylation in Cardiovascular Oxidative and Inflammatory Injury

Available evidence indicates that SUMOylation regulates several processes central to cardiovascular pathophysiology. SUMO signaling influences mitochondrial dynamics, endothelial nitric oxide homeostasis, metabolic transcription, inflammasome activation, and vascular remodeling [34,35,36,37,38,39,40,41,42,43,49,50,51,52,53,54,55,56,57,58,59,60,61,62,63,64]. In vascular cells, PIAS proteins and SENPs regulate endothelial and vascular smooth-muscle phenotypes through eNOS, ERK5, p53, GATA2, PPARγ, and related transcriptional pathways [34,35,56,59,60,61,62,63,64,65]. In immune cells, SUMOylation regulates the threshold and duration of NLRP3 and NF-κB activation, thereby linking post-translational control to inflammation and cell death [41,42,43,66,67,68,69,70,71,72,73,74,75,76].

However, the mechanistic strength of evidence differs substantially among these pathways. Some studies rely primarily on manipulation of SUMO pathway components or changes in global SUMO-conjugate abundance, whereas others demonstrate SUMOylation of a defined substrate and, in fewer cases, identify specific acceptor residues. Moreover, apparently divergent effects of SUMOylation have been reported for some substrates under different experimental conditions. These differences may reflect variation in SUMO paralogue, modification site, enzyme context, cell type, subcellular compartment, or stress intensity and duration. The following sections therefore focus on the best-characterized cardiovascular SUMO-regulated mechanisms while distinguishing established substrate-level mechanisms from broader pathway-level associations [18,21,22,23,24,25,26,27,28].

## 3. Molecular Mechanisms Linking SUMOylation to Cardiovascular Stress

Cardiovascular stress is rarely driven by a single pathway. Oxidative stress, mitochondrial dysfunction, calcium dysregulation, inflammatory signaling, and regulated cell death develop in parallel and reinforce one another. Within this network, SUMOylation modifies the stress response in a substrate-, enzyme-, cell type-, and disease stage-dependent manner. The most convincing evidence supports roles for SUMOylation in redox-responsive remodeling of the SUMO machinery, mitochondrial and calcium homeostasis, metabolic adaptation, and inflammatory licensing.

### 3.1. Organelle Homeostasis and Mitochondrial Quality Control

Mitochondrial dynamics and quality control are central determinants of cardiomyocyte survival under stress. Fission, fusion, mitophagy, and mitochondrial proteostasis are all vulnerable to redox perturbation and can be reshaped by SUMOylation [36,37,38,39,40,47,77]. The best-characterized mitochondrial axis is the SENP3–Drp1 module, which couples oxidative stress to mitochondrial recruitment of Drp1, mitochondrial fragmentation, and cell death [38]. In this context, SENP3-mediated regulation of Drp1 provides an example of how redox-dependent changes in a SUMO protease can influence mitochondrial remodeling. However, the direction of Drp1 regulation by SUMOylation is not uniform across experimental systems. Studies have reported both effects associated with Drp1 deSUMOylation and Drp1 SUMOylation on mitochondrial remodeling, suggesting that the SUMO paralogue involved, stress condition, and experimental manipulation may influence the outcome [38,39]. Moreover, the specific SUMO-acceptor sites responsible for the cardiovascular effects of the SENP3–Drp1 axis remain incompletely resolved.

SUMO-dependent mitochondrial regulation extends beyond a single fission factor. MAPL-mediated SUMOylation of Drp1 stabilizes an endoplasmic reticulum–mitochondrial platform involved in cell death [39]. SENP5 has also been implicated in mitochondrial remodeling and cardiomyopathy, whereas SENP1-linked regulation contributes to mitochondrial abnormalities in the myocardium [40,78]. These findings suggest that SUMO signaling organizes mitochondrial dynamics at several levels rather than acting through a single fission regulator. Nevertheless, the extent to which these effects reflect direct substrate modification versus broader changes in the SUMO machinery varies among experimental models.

DUSP6 SUMOylation further demonstrates the interaction between SUMOylation and phosphorylation. By regulating Drp1 dephosphorylation, DUSP6 SUMOylation can protect cells from oxidative damage and influence mitochondrial fragmentation [37]. Reviews and proteomic studies suggest that mitophagy-related pathways may also be subject to SUMO control [36,47,77]. These observations support a broader role for SUMO signaling in mitochondrial quality control while highlighting the need for additional substrate- and site-resolved studies in cardiovascular models.

### 3.2. Calcium Handling and Metabolic Adaptation

SUMOylation also regulates calcium handling and metabolic adaptation. The strongest cardiovascular example is SUMO1-dependent regulation of SERCA2a, which supports sarcoplasmic-reticulum calcium reuptake, contractile function, and cardiac performance in heart failure [49]. Restoration of this axis has provided a proof-of-concept for substrate-directed SUMO modulation. Small-molecule enhancement of SERCA2a SUMOylation improves cardiac function, and SUMO1 gene transfer has produced beneficial effects in experimental large-animal heart failure models [49,50,51].

Compared with several other cardiovascular SUMO-regulated pathways, the SUMO1–SERCA2a axis has relatively strong substrate-level mechanistic support. SUMO1 has been reported to modify SERCA2a at K480 and K585, and mutation of these residues markedly reduces SERCA2a SUMOylation, ATP-binding capacity, ATPase activity, and protein stability [49]. These findings provide site-resolved evidence linking a defined SUMO modification to calcium-handling function. Consistent with this mechanism, increasing SUMO1 availability has been associated with improved SERCA2a function and cardiac performance in experimental heart failure models [49,50]. Nevertheless, the relative contribution of K480 versus K585 in vivo, as well as the extent to which these modifications are dynamically regulated across different cardiovascular stress states, remains incompletely defined [49,79]. Thus, the beneficial effects associated with increasing SUMO1 availability should not be interpreted as evidence that global enhancement of SUMOylation is uniformly beneficial across cardiovascular substrates [44,45].

SUMOylation also intersects with metabolic reprogramming through SIRT1, PGC-1α, PPARα, C/EBPβ, TEAD1, and related transcriptional regulators [52,53,54,55,80,81,82]. These pathways coordinate oxidative metabolism, mitochondrial biogenesis, hypertrophic growth, and stress adaptation. In chronic cardiovascular disease, where energetic reserve is progressively depleted, SUMO-dependent regulation of metabolic transcription factors may be as important as direct control of contractile proteins.

In vascular disease, PIAS1- and SENP-dependent regulation of PPARγ-related networks links SUMO signaling to lipid handling, inflammation, and structural remodeling [34,56,64]. Together, these findings support a model in which SUMOylation integrates calcium handling, mitochondrial metabolism, and inflammatory transcription rather than regulating these processes independently.

Beyond intracellular protein-modification pathways, emerging bioelectronic and biomaterial-based approaches further illustrate the multilayered nature of cardiac calcium and electrical dysfunction. Electrically conductive collagen–PEDOT hydrogels have been reported to improve calcium handling, contractility, and conduction properties of human induced pluripotent stem cell-derived cardiomyocytes and to reduce ventricular tachycardia in a post-myocardial-infarction mouse model [83]. PEDOT microstructured electrodes have also been developed as cytocompatible bioelectronic platforms with defined electrochemical and mechanical properties [84]. Although these approaches are mechanistically distinct from SUMOylation, they highlight the broader therapeutic landscape through which cardiac calcium handling and electrical function can be modulated.

### 3.3. Inflammatory Licensing and Regulated Cell Death

SUMOylation regulates inflammatory licensing rather than simply suppressing inflammation. NLRP3 inflammasome activation is a major point of convergence between oxidative stress, mitochondrial dysfunction, and sterile inflammation [71,72,85]. SUMO-dependent regulation of NLRP3 has been reported in several studies: SUMO1 can modify NLRP3, whereas SENP3-mediated deSUMOylation can alter inflammasome activation and macrophage responses [41,57]. TRIM28-mediated SUMOylation stabilizes NLRP3 and facilitates inflammasome activation [69]. Thus, the functional consequences of NLRP3 SUMOylation depend on the modifying enzyme, SUMO paralogue, substrate site, and cellular context.

Importantly, NLRP3 provides a clear example of how the functional consequences of SUMOylation cannot be inferred from global SUMO-conjugate levels alone. Site-resolved studies have identified distinct SUMO-dependent regulatory events with opposing effects on NLRP3 activation. SUMO1 has been reported to modify NLRP3 at K204, and mutation of this residue reduces NLRP3 SUMOylation and attenuates ASC oligomerization, inflammasome activation, and IL-1β secretion; conversely, SENP3-mediated deSUMOylation suppresses these responses [41]. In contrast, K689 has been identified as a SUMO-acceptor site in a study showing that MAPL/MUL1-dependent NLRP3 SUMOylation restrains inflammasome activation, whereas loss of this SUMO-dependent regulation enhances NLRP3 activation [70]. Thus, the directionality of NLRP3 regulation is not uniform and may depend on the SUMO paralogue, acceptor site, E3 ligase or SUMO protease, and experimental context.

TRIM28-mediated SUMOylation provides a further example of substrate-level regulation. TRIM28 promotes SUMO1/2/3 modification of NLRP3, reduces NLRP3 ubiquitination and proteasomal degradation, and thereby increases NLRP3 abundance and inflammasome activation [69]. However, the relevant SUMO-acceptor residues underlying the TRIM28 effect have not been resolved. Moreover, most mechanistic studies of NLRP3 SUMOylation have been performed in macrophages or other non-cardiovascular models. Therefore, although these studies establish direct regulation of NLRP3 at the substrate level, the extent to which the identified site-specific mechanisms operate similarly in cardiomyocytes or vascular cells remains uncertain. Global changes in SUMO-conjugate abundance should likewise not be interpreted as evidence for a uniform effect on NLRP3 activity.

Canonical NF-κB signaling is also shaped by SUMOylation at several nodes, with evidence ranging from site-specific substrate modification to pathway-level manipulation. SUMO1 modification of IκBα at K21 can prevent ubiquitin-dependent degradation of IκBα and thereby restrain NF-κB activation [68,86]. In contrast, SUMO1 modification of NEMO/IKKγ at defined lysine residues, including K277 and K309 in the context of genotoxic stress, promotes the nuclear signaling events required for NF-κB activation [67]. These findings illustrate that SUMOylation can exert opposing effects within the same inflammatory pathway depending on the substrate and signaling context. However, these site-resolved mechanisms were established primarily in non-cardiovascular experimental systems, and their relevance to cardiovascular stress remains less well defined. Accordingly, changes in global SUMOylation or manipulation of SUMO enzymes should be distinguished from direct evidence for modification of individual NF-κB components.

SUMOylation also intersects with apoptosis, pyroptosis, and ferroptosis. These forms of regulated cell death contribute to myocardial injury, vascular inflammation, and adverse remodeling [71,72,73,74,75,76,85,87,88]. In these settings, evidence for SUMO involvement is more heterogeneous than for the site-resolved NLRP3–SUMO and SERCA2a–SUMO axes. Some studies demonstrate changes in specific SUMO-regulated proteins or SUMO enzyme activity, whereas others rely primarily on manipulation of SUMO pathway components or changes in global SUMO-conjugate levels [89,90]. Consequently, SUMO-dependent effects on regulated cell death should be interpreted cautiously, particularly when direct substrate modification or acceptor sites have not been established. Further studies combining site-resolved SUMOylation analysis, substrate-specific SUMO-site mutants, and functional rescue experiments in cardiovascular models will be needed to determine whether these mechanisms represent direct substrate regulation or secondary consequences of broader remodeling of the SUMO system [44,47,48].

## 4. Cell- and Disease-Specific SUMO Signaling in Cardiovascular Pathology

**Figure 2:**
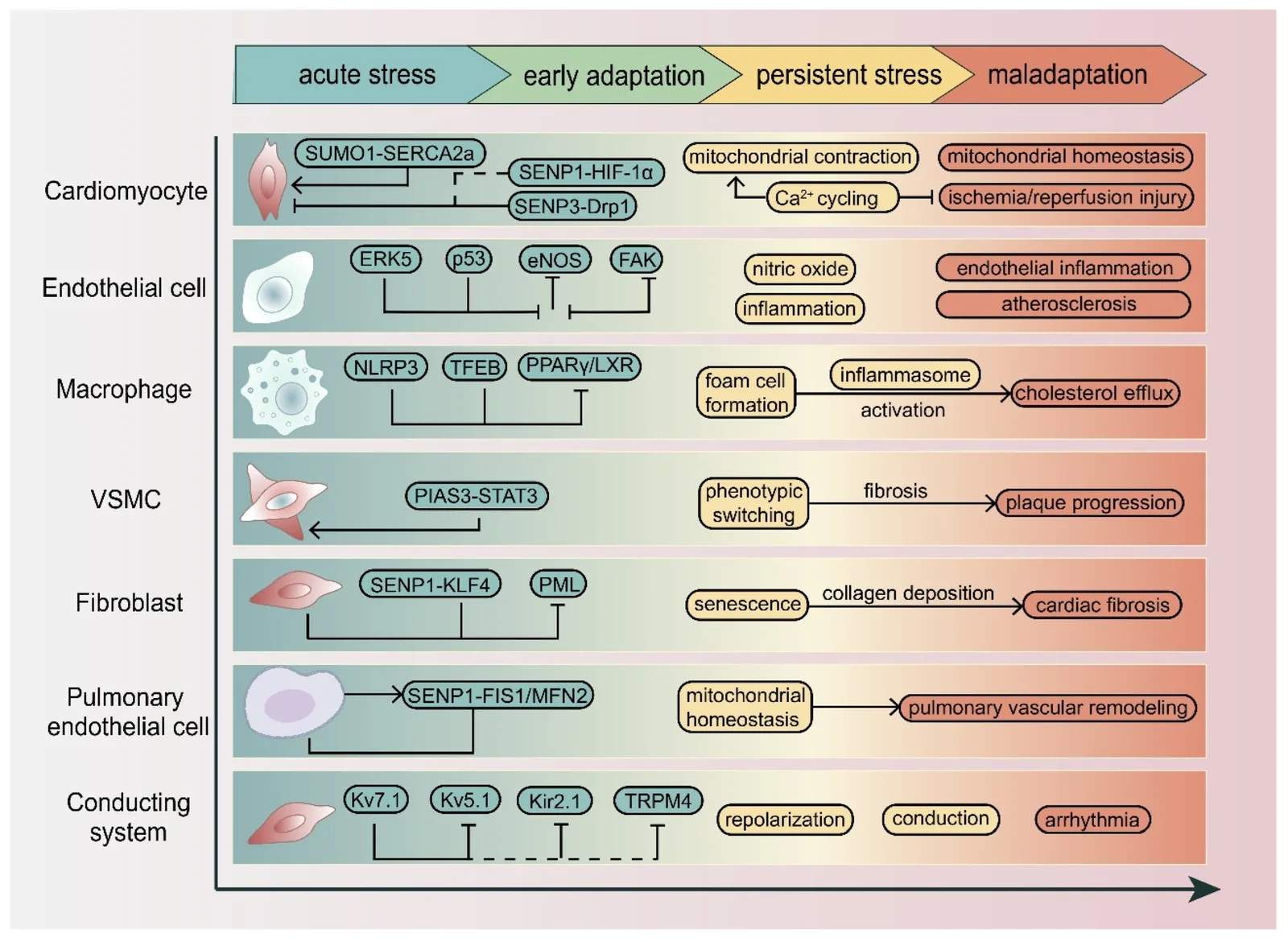
Integration of SUMO signaling across cardiovascular cell types and disease stages.

The consequences of SUMOylation are determined not only by the modified protein but also by the cell type in which the modification occurs. Cardiomyocytes, endothelial cells, vascular smooth-muscle cells, fibroblasts, and lesional myeloid cells experience distinct metabolic, hemodynamic, and inflammatory pressures. Meaningful interpretation of SUMO signaling in CVD therefore requires integration of substrate-specific mechanisms with the cellular microenvironment. The proposed integration of SUMO signaling across cardiovascular cell types and disease stages is summarized in Figure 2.

Table 1 summarizes the major SUMO-regulatory axes, their key molecular mechanisms, functional outcomes, and associated cardiovascular disease contexts, providing an overview of the evidence discussed in the following subsections.

SUMO signaling in cardiovascular and pulmonary vascular pathology is integrated along two axes: disease progression (acute stress → early adaptation → persistent stress → maladaptive remodeling) on the horizontal axis and major cell types on the vertical axis, highlighting stage- and cell-specific effects. In cardiomyocytes, SUMO1–SERCA2a and SENP3–Drp1 link SUMOylation to Ca^2+^ handling and mitochondrial homeostasis; in endothelial cells, ERK5 and eNOS couple SUMO signaling to NO production and vascular inflammation; in macrophages, NLRP3 and PPARγ/LXR connect it to foam-cell formation and cholesterol efflux; in vascular smooth muscle cells, the PIAS3–STAT3 axis is implicated in phenotypic switching; fibroblast SENP1–KLF4 and PML modules, together with FIS1/Drp1 SUMO regulation and mitochondrial dynamics in pulmonary endothelial cells, are implicated in fibrosis and pulmonary vascular remodeling, respectively; and in the cardiac conduction system, ion channels including Kv7.1 and Kir2.1 modulate repolarization and arrhythmia susceptibility. The color gradient indicates the conceptual transition from adaptive states toward persistent stress and maladaptive remodeling.

Abbreviations: SUMO, small ubiquitin-like modifier; SERCA2a, sarco/endoplasmic reticulum Ca^2+^-ATPase 2a; SENP3, sentrin/SUMO-specific protease 3; Drp1, dynamin-related protein 1; ERK5, extracellular signal-regulated kinase 5; eNOS, endothelial nitric oxide synthase; NO, nitric oxide; NLRP3, NLR family pyrin domain containing 3; PPARγ, peroxisome proliferator-activated receptor γ; LXR, liver X receptor; VSMCs, vascular smooth muscle cells; PIAS3, protein inhibitor of activated STAT3; STAT3, signal transducer and activator of transcription 3; SENP1, sentrin/SUMO-specific protease 1; KLF4, Krüppel-like factor 4; PML, promyelocytic leukemia protein; FIS1, fission 1; MFN2, mitofusin 2; Kv7.1, voltage-gated potassium channel 7.1; Kir2.1, inward rectifier potassium channel Kir2.1.

### 4.1. Atherosclerosis: Disturbed Flow, Endothelial Dysfunction, and Lesional Inflammation

Atherosclerosis is informative because SUMO signaling is altered across several cell populations within the same lesion. In endothelial cells, disturbed flow promotes inflammatory activation through ERK5 SUMOylation and p53-related signaling, suppressing anti-inflammatory transcriptional programs [59,61,62]. SENP2 acts as an important counter-regulatory factor, and inhibition of SENP2 by disturbed-flow signaling permits persistent pro-atherogenic responses [60].

SUMOylation also contributes to endothelial nitric oxide homeostasis. PIAS1-mediated SUMOylation of eNOS supports endothelial function and vascular homeostasis [35]. Additional SUMO-dependent transcriptional pathways influence vascular remodeling and smooth-muscle phenotypic behavior [63,64,65]. In plaque macrophages, SENP3–NLRP3 signaling links ox-LDL exposure to inflammasome activation and foam-cell formation [57]. TFEB SUMOylation further connects lysosomal dysfunction, impaired cholesterol handling, and foam-cell biology [58].

The broader vascular wall also matters. Graft arteriosclerosis and lipid-driven vascular remodeling reveal that SUMO signaling can shape endothelial activation, immune-cell recruitment, lipid metabolism, and vascular structure [34,60,63,64,111]. Atherosclerosis should therefore be understood as a lesion environment in which SUMO-dependent signaling coordinates endothelial, immune, and smooth-muscle programs simultaneously.

### 4.2. Myocardial Ischemia/Reperfusion Injury

Myocardial I/R injury provides a clinically relevant setting in which oxidative stress, calcium overload, mitochondrial dysfunction, and sterile inflammation occur within a short time window. Current evidence supports dynamic remodeling of SUMO signaling during I/R rather than a static change in a single pathway [4,77,87,96,97,98,99,100,103].

Several SUMO-dependent mechanisms appear adaptive during early stress. SUMO2-dependent modification of nuclear γ-actin supports DNA damage repair and limits ischemia-induced genotoxic injury [99]. Upregulation of SUMO2 and global protein SUMOylation has also been associated with cardioprotection in experimental I/R models [100]. SENP1-dependent HIF-1α signaling contributes to myocardial protection during I/R, whereas Nrf2 deSUMOylation has been linked to reduced ferroptotic injury [87,96]. These findings suggest that early ischemia and reperfusion may activate coordinated repair, antioxidant, and survival programs.

The SENP3–Drp1 axis illustrates this context dependence. In one experimental setting, SENP3-mediated deSUMOylation of Drp1 promotes its interaction with the mitochondrial fission factor Mff, increasing mitochondrial recruitment of Drp1 and cytochrome c release during reoxygenation [38]. In contrast, studies of myocardial I/R have reported a protective role for SENP3, with ischemia-associated loss of cytosolic SENP3 and SENP3 knockdown increasing cell death after reperfusion [98]. These observations do not necessarily establish intrinsically opposing functions of SENP3 across all I/R contexts. The two studies differed in cellular and experimental context: the Drp1 study primarily used HEK293/HeLa cell systems subjected to oxygen-glucose deprivation/reoxygenation and manipulated the SENP3–Drp1–Mff axis, whereas the myocardial I/R study examined Langendorff-perfused hearts and cardiomyocyte-like H9C2 cells [38,98]. In addition, Drp1 can be modified by different SUMO paralogues, and the functional consequences of these modifications are not necessarily equivalent [38,115]. Indeed, SUMO1-dependent Drp1 modification has been associated with protection in a zinc-treated I/R model, further illustrating that the consequence of Drp1 SUMOylation can depend on the modifying pathway and experimental context [97].

Thus, the relevant variable is not simply whether SENP3 or Drp1 SUMOylation is increased or decreased, but which enzyme–substrate interaction is altered, in which compartment, and at which stage of the I/R response [38,98,116,117]. During ischemia and reperfusion, changes in redox state, cellular energy status, protein localization, and protein–protein interactions may alter substrate accessibility and thereby reshape the functional consequences of SUMO modification [38,97,98,116]. The distinction is particularly important when extrapolating from transformed cell lines or isolated cardiomyocyte models to intact myocardium, where multiple cell types and spatially heterogeneous stress environments coexist [44,97,98].

Overall, I/R provides a useful example of why SUMO signaling should be interpreted as a dynamic and spatially organized regulatory system. Early adaptive responses, reperfusion-associated oxidative stress, and subsequent injury may engage different SUMO substrates or enzyme pools. The apparent divergence among SENP3-, Drp1-, and SUMO2/3-dependent observations is therefore better interpreted in terms of temporal, compartmental, substrate-, SUMO-paralogue-, and model-dependent regulation rather than as evidence for a single universal direction of SUMO signaling [38,77,97,98,99].

### 4.3. Cardiac Remodeling and Heart Failure

Chronic cardiac remodeling differs from I/R injury in that the dominant stressors are persistent mechanical load, neurohormonal activation, metabolic dysfunction, fibrosis, and low-grade inflammation. The central issue is not a global increase or decrease in SUMOylation but progressive dysregulation of specific adaptive pathways [45,49,52,53,54,55,78,80,81,82,91,92,93,95,118,119].

The best-established axis remains SERCA2a SUMOylation, whose disruption is associated with heart failure [49,50,51,81,93]. Metabolic remodeling provides a second dimension: SUMOylation of SIRT1, regulation of PGC-1α-related transcription, and modification of TEAD1, C/EBPβ, and β-catenin influence hypertrophy, fibrosis, mitochondrial metabolism, and energetic failure [45,52,53,54,55,78,80,81,82,92,93,95,118,119].

SENP1 also modulates pressure overload-induced remodeling through STAT3-related signaling [91]. Other implicated regulators include HSF2, PML, SENP5, and XBP1; notably, XBP1 SUMOylation regulates the miR-146a axis in diabetic cardiac dysfunction [78,81,82,92,93,95]. Together, these data suggest that chronic cardiac stress is associated with failure to preserve coordinated calcium, mitochondrial, and metabolic adaptation rather than disruption of a single isolated pathway.

### 4.4. Arrhythmia, Pulmonary Vascular Disease, and Emerging Contexts

SUMOylation influences cardiac electrophysiology beyond the remodeling phenotype: modification of Kv1.5, Kv7.1, and Kir2.1 alters channel gating and may contribute to arrhythmia susceptibility [104,105,106]. Experimental studies implicate SUMO-dependent regulation of FIS1 and Drp1 in pulmonary vascular remodeling [114,120]. Transcriptomic and bioinformatic analyses have further suggested broader links between SUMO-related genes and pulmonary arterial hypertension [121], extending SUMO signaling from myocardium and systemic arteries to the pulmonary circulation. Peripheral arterial disease and viral myocarditis provide additional cardiovascular contexts, although SUMO-dependent mechanisms remain poorly characterized [109,122].

## 5. The SUMO Rheostat Model

The studies reviewed above indicate that SUMOylation cannot be interpreted as uniformly protective or pathogenic in cardiovascular disease. Its functional consequences depend on the integrated state of the stressed cell, including the source, intensity, and duration of stress; subcellular redox conditions; antioxidant buffering capacity; SUMO paralogue availability; substrate abundance and accessibility; and the activity and localization of SUMO E3 ligases and SENPs [25,26,27,28,29,30,31,32,33,44]. These variables determine not only the overall extent of SUMO conjugation but also which substrates are modified, where modification occurs, and how long the resulting regulation persists.

**Figure 3:**
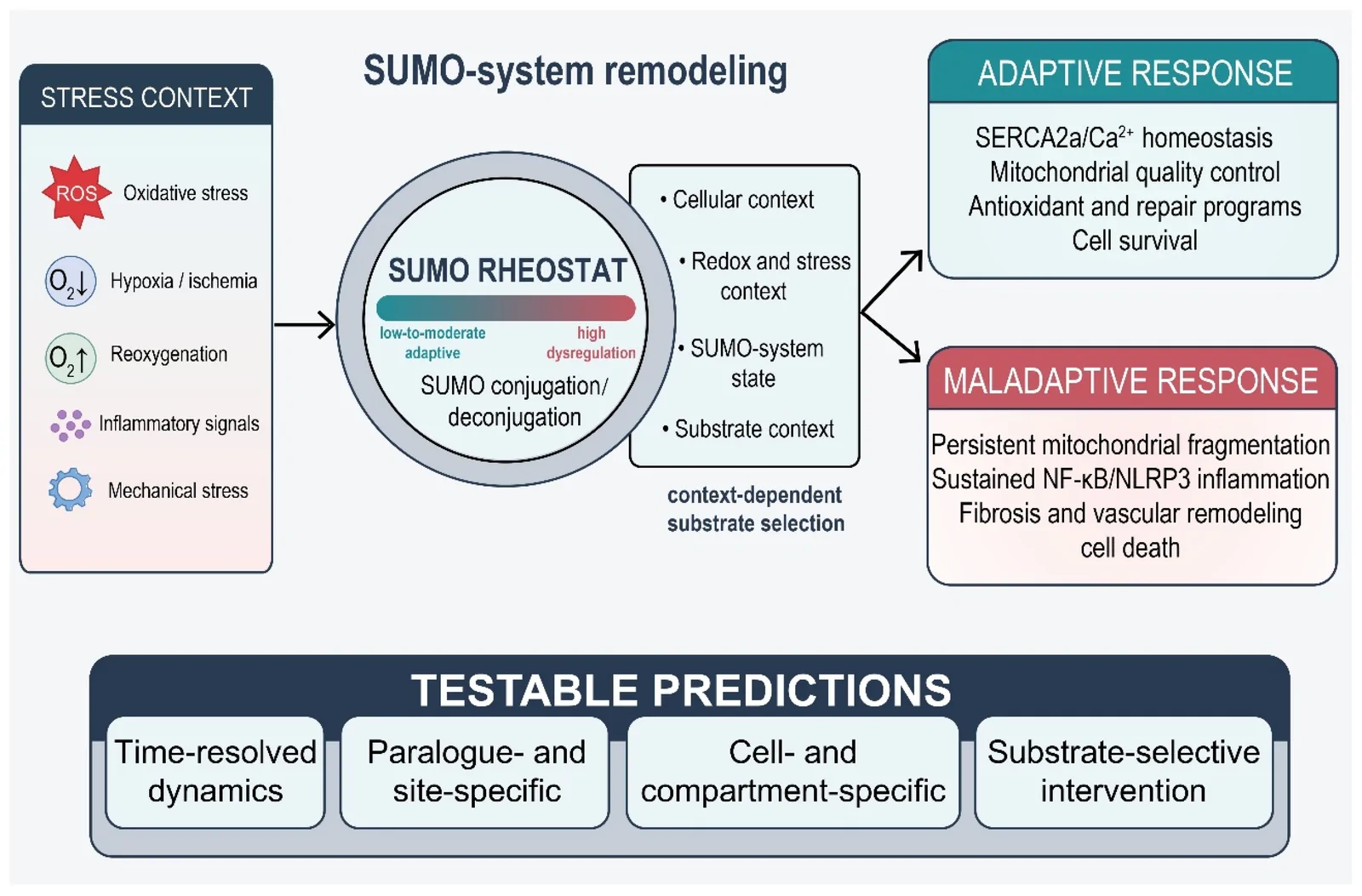
Mechanistic SUMO rheostat model in cardiovascular stress.

Building on this context-dependent view, we propose a SUMO rheostat model in which SUMO signaling dynamically modulates the amplitude, timing, and spatial distribution of cellular stress responses rather than functioning as a binary protective or pathogenic switch. As summarized in Figure 3, the integrated state of the cell may redirect SUMO signaling toward distinct adaptive or maladaptive outcomes. The model is intended as a conceptual and testable framework for integrating apparently divergent observations across cardiovascular cell types and disease settings, rather than as an established quantitative relationship between stress intensity and global SUMOylation.

SUMOylation is proposed as a context-dependent molecular rheostat that modulates cardiovascular stress responses rather than acting as a uniformly protective or pathogenic pathway. Representative stress inputs, including oxidative stress, hypoxia/ischemia, reoxygenation, inflammatory signals, and mechanical stress, may remodel SUMO signaling through changes in SUMO conjugation/deconjugation and regulatory enzyme activity.

The central panel highlights four contextual determinants of SUMO substrate regulation: cellular context, redox/stress context, SUMO-system state, and substrate context. Together, these factors may determine substrate selection, modification, localization, and persistence, thereby shaping adaptive or maladaptive outcomes. Adaptive responses include maintenance of Ca^2+^ homeostasis, mitochondrial quality control, antioxidant and repair programs, and cell survival, whereas maladaptive responses include mitochondrial fragmentation, NF-κB/NLRP3-associated inflammation, fibrosis and vascular remodeling, and cell death. The lower panel summarizes testable predictions, including time-, SUMO-paralogue-, site-, cell-, and compartment-specific regulation and opportunities for substrate-selective intervention. Unlike Figure 1, which introduces the rheostat concept and canonical SUMOylation cycle, Figure 3 focuses on the contextual determinants and downstream consequences of SUMO signaling. The model is conceptual and testable rather than a quantitative relationship between stress intensity and global SUMOylation.

### 5.1. Redox-Sensitive Remodeling of the SUMO System

A central premise of the rheostat model is that the SUMO machinery itself is responsive to redox stress. Oxidative conditions can alter the activity of SUMO-conjugating enzymes and the stability, localization, or activity of SENPs, thereby changing the cellular capacity for SUMO conjugation and deconjugation rather than simply modifying downstream substrates [25,29,30,31,32,33]. This provides a mechanistic basis for considering SUMOylation as part of the cellular stress-response machinery.

SENP3 provides an illustrative example of this redox sensitivity. Under mild oxidative stress, SENP3 can be stabilized and promote transcriptional adaptation through deSUMOylation of regulatory cofactors such as p300 [30,31,32]. However, the functional consequences of SENP3 activity are highly context-dependent. In cardiovascular and other stress models, SENP3-dependent deSUMOylation has been linked to mitochondrial dynamics, inflammatory signaling, and cell survival, with effects that can vary according to the substrate and experimental context [30,31,38,97,98]. Thus, the SUMO system functions as an active component of the cellular redox-response machinery, dynamically reconfiguring SUMOylation in a context- and time-dependent manner. This redox responsiveness also creates a therapeutic opportunity, but one that may require temporally and contextually selective intervention rather than constitutive modulation of the pathway.

### 5.2. Substrate-, SUMO-Paralogue-, and Cell-Dependent Regulation

Distinct SUMO paralogues and regulatory enzymes can produce different, and in some cases opposing, functional outcomes within the same signaling pathway: SUMO1–IκBα restrains canonical NF-κB, whereas SUMO2/3–ubiquitin chains and NEMO modification facilitate activation in selected contexts [66]; NLRP3 can be restrained or activated depending on the modifying enzyme, SUMO paralogue, and deSUMOylase [69,70]. Importantly, the functional output also depends on substrate accessibility. Enzyme abundance alone does not determine which substrates are modified because substrate localization, protein abundance, local protein–protein interactions, compartmental redox conditions, and the timing of stress can constrain enzyme–substrate encounters. Thus, the same change in SENP or E3-ligase activity may have different consequences when substrate pools are redistributed or when competing substrates become preferentially accessible [46].

Cellular context is equally decisive—calcium handling and mitochondria in cardiomyocytes [40,49,97], ERK5/p53 in disturbed-flow endothelium [59,60,61,62], lipid handling and inflammasomes in macrophages [57,72,73,74], and phenotypic switching in smooth muscle [65]. These differences caution against interpreting pathway output from any single enzyme or global SUMO readout alone and support the need for cell type-resolved analysis to identify distinct SUMO-regulatory networks. Importantly, apparently similar changes in global SUMOylation may therefore have distinct functional consequences across cell types or disease stages.

### 5.3. Evidence Boundaries and Unresolved Contradictions

The SUMO rheostat model is a conceptual synthesis rather than a validated predictive framework. Several important uncertainties therefore limit the extent to which the model can currently be generalized.

First, quantitative relationships between ROS burden and SUMO conjugation have not been established reproducibly across cardiovascular cell types. It remains unclear whether SUMO1 and SUMO2/3 exhibit distinct stress-response relationships in vivo or whether apparent differences primarily reflect substrate availability and local enzyme activity [25,44].

Second, stress duration and temporal sequence are likely to be important determinants of SUMO output. Acute oxidative stress, prolonged ischemia, early reperfusion, and chronic remodeling represent biologically distinct states rather than simply different points on a single stress-intensity scale. Enzyme abundance, catalytic activity, substrate localization, and protein–protein interactions can change during these transitions. Consequently, a SUMO-dependent mechanism that is adaptive during an early response may not remain dominant during prolonged or spatially redistributed stress [97,98,116].

Third, subcellular compartment and substrate accessibility may provide a mechanistic explanation for apparently divergent effects of the same SUMO regulator. SENPs and E3 ligases can occupy distinct cellular compartments, while substrates can undergo stress-dependent redistribution or changes in abundance. Thus, an alteration in total SENP activity does not necessarily predict the modification state of every substrate. The SENP3–Drp1 example is particularly informative: deSUMOylation of Drp1 can facilitate Mff-dependent mitochondrial recruitment and cell death in a reoxygenation model, whereas loss of cytosolic SENP3 has been associated with protection in myocardial I/R [38,98]. These findings may reflect differences in the accessible SENP3 pool, the dominant substrate and interacting partners, and the timing of the perturbation rather than a universal reversal of SENP3 function.

Fourth, differences among SUMO paralogues and their modification sites further complicate interpretation. Drp1 can be modified by SUMO1 and SUMO2/3, and available evidence indicates that these modifications can influence mitochondrial localization through distinct mechanisms. Similar complexity may apply to other cardiovascular substrates, but endogenous site-specific measurements remain limited. Global SUMO1 or SUMO2/3 conjugate measurements therefore provide insufficient resolution to determine which functional SUMO events are responsible for a given phenotype [44,47,77].

Fifth, experimental model differences must be considered when apparently contradictory results are compared. Transformed cell lines, primary cardiomyocytes, cardiomyoblasts, isolated perfused hearts, and intact animal models differ in cellular composition, metabolic state, redox buffering, substrate abundance, and tissue architecture [44,98]. Even within I/R studies, ischemic duration, reperfusion interval, preconditioning, injury severity, and the timing and mode of genetic or pharmacological intervention can alter the dominant stress-response pathway [97,117]. Therefore, observations made in one experimental system should not automatically be interpreted as a general property of the SUMO pathway in cardiovascular disease.

These considerations argue against resolving divergent observations by assigning a single protective or pathogenic direction to a SUMO enzyme or substrate. Instead, the relevant question is which SUMO-regulated interaction is altered, in which compartment, at what stage of stress, and under which experimental or disease context. Resolving these uncertainties will require time-resolved, endogenous, site-specific, and SUMO-paralogue-resolved measurements combined with cell type- and compartment-specific perturbation strategies [44,47,48].

### 5.4. Testable Predictions of the SUMO Rheostat Model

The rheostat model generates several experimentally testable predictions.

First, SUMO signaling should exhibit temporal dynamics rather than a simple increase-or-decrease response. The pattern of SUMO conjugation and deconjugation should change across the transition from acute stress to adaptation and, under persistent stress, to maladaptive injury. Time-resolved analyses of cardiac ischemia–reperfusion already provide evidence that SUMOylation is dynamically remodeled during stress [77].

Second, SUMO1 and SUMO2/3 should display partially distinct stress-response patterns. Differences should become apparent at the level of individual substrates, modification sites, and subcellular compartments rather than necessarily at the level of total cellular SUMO abundance [44,47,77].

Third, the same systemic stressor should produce distinct SUMO-regulatory responses across cardiovascular cell types. Cardiomyocytes, endothelial cells, vascular smooth muscle cells, fibroblasts, and lesional myeloid cells are predicted to differ in substrate availability, SUMO enzyme activity, and downstream functional consequences.

Fourth, SUMO responses should be spatially heterogeneous. Nuclear, mitochondrial, cytoplasmic, and membrane-associated SUMO pools may undergo different degrees or kinetics of remodeling during the same stress exposure. Such compartmentalization may help explain why measurements of total cellular SUMOylation can fail to predict functional outcomes.

Fifth, selective manipulation of individual SUMO–substrate relationships may offer greater therapeutic specificity than global modulation of the SUMO cascade while preserving unrelated SUMO-dependent functions.

Testing these predictions will require integration of time-resolved SUMO proteomics, endogenous site-specific SUMOylation analysis, SUMO-paralogue-resolved approaches, cell type-specific genetic models, and functional phenotyping. Depending on the disease context, relevant readouts should include mitochondrial respiration, calcium handling, vascular reactivity, inflammatory and inflammasome activity, cell survival, and tissue remodeling [44,47,77,123,124].

The rheostat model should therefore be viewed as a testable framework for organizing current evidence and generating experimentally falsifiable predictions, rather than as an established universal principle of cardiovascular SUMO biology. Its ultimate value will depend on whether future studies can demonstrate reproducible relationships between stress dynamics, SUMO substrate selection, cellular context, and cardiovascular outcomes.

## 6. Translational Implications and Future Directions

### 6.1. From Global SUMO Modulation to Precision Cardiovascular Targeting

The SUMO pathway is pharmacologically tractable, but its broad physiological functions impose substantial limitations on systemic intervention. SUMOylation regulates DNA repair, chromosome segregation, proteostasis, transcriptional control, innate immune signaling, and cellular stress adaptation [18,19,20,21,22,23,24,26,27]. Broad disruption of the pathway may therefore produce substantial toxicity.

Small-molecule inhibition of the SUMO E1 enzyme has demonstrated that the SUMO cascade can be manipulated pharmacologically. Oncology-oriented inhibitors such as TAK-981/Subasumstat have also provided important information regarding pharmacodynamic activity, therapeutic windows, and potential systemic toxicity [125,126,127,128,129]. However, these agents were developed primarily for cancer treatment and should not be directly extrapolated to cardiovascular therapy.

A more promising strategy is to selectively manipulate beneficial SUMO–substrate axes. The strongest proof of concept remains the SUMO1–SERCA2a pathway, in which restoring adaptive SUMOylation improves cardiac function [49,50,51]. Transient inhibition of specific SUMO enzymes during pathological redox stress may also be beneficial, as suggested by experimental SAE1-focused intervention in pressure overload remodeling [130]. Given the context dependence of SUMO signaling described above, future cardiovascular therapies will likely require selectivity at one or more levels: substrate selectivity, SENP or E3-ligase selectivity, SUMO-paralogue selectivity, cell type-specific delivery, and disease-stage- or time-specific administration. Such approaches may help preserve physiological SUMO-dependent functions while selectively modifying disease-relevant SUMO signaling.

### 6.2. Biomarker Development: Biological Promise but Limited Clinical Readiness

Biomarker development remains exploratory. Tissue-level evidence is currently strongest for disease-relevant SUMO-regulatory axes, whereas circulating measurements may not adequately capture signaling within cardiomyocytes, endothelial cells, vascular smooth muscle cells, or lesional macrophages. Moreover, transcriptomic changes in SUMO1, UBC9, or SENPs do not necessarily reflect the abundance of endogenous SUMO conjugates or substrate-specific SUMOylation activity [47,77,131]. Future biomarker strategies will therefore likely need to integrate circulating injury and inflammatory markers with imaging-based functional assessment, cell-specific SUMO signatures, and longitudinal phenotyping. The current evidence hierarchy and translational status of the major SUMO-related cardiovascular findings are summarized in Table 2.

### 6.3. Future Perspective

The central translational question is not whether SUMOylation should be globally increased or decreased, but which SUMO-regulated substrate should be modified, in which cell type, at what disease stage, and for how long. For chronic heart failure, substrate-specific enhancement of calcium handling and metabolism is more realistic than global SUMO activation; for atherosclerosis, endothelial-targeted restoration of protective SUMO signaling may be preferable to systemic immunomodulation; for myocardial I/R, temporally restricted intervention is required because SUMO signaling differs between ischemia, early reperfusion, and late remodeling. Notably, the evidence base remains centered on the heart and large vessels; peripheral arterial disease is a major gap, and SUMO-dependent regulation of angiogenesis and collateral formation warrants systematic study [122]. The priority is not broader pathway modulation but deeper resolution of spatial, temporal, and substrate-specific SUMO biology.

## 7. Conclusions

SUMOylation is a dynamic post-translational regulatory system connecting cellular redox status to mitochondrial homeostasis, calcium handling, inflammatory signaling, and stress adaptation in cardiovascular disease. Current evidence does not support classifying SUMOylation, individual SUMO paralogues, or SENP enzymes as uniformly protective or pathogenic: outcomes are determined by substrate, SUMO paralogue, enzyme, subcellular compartment, cell type, and disease stage. Synthesizing these observations, the SUMO rheostat framework models SUMOylation as a modulator of the magnitude, duration, and localization of stress responses; its validation will require endogenous, site-specific, SUMO-paralogue-resolved, cell-resolved, and time-resolved studies. The translational challenge is to identify actionable SUMO–substrate axes in defined cells and disease stages, using rigorous SUMO proteomics, spatial and single-cell approaches, cell type-specific models, and selective delivery—precision strategies that preserve adaptive SUMO responses while limiting pathological remodeling in heart failure, atherosclerosis, and myocardial I/R injury.

## Figures and Tables

**Table 1:** SUMOylation-associated regulatory axes and functional outcomes in cardiovascular diseases.

Cardiovascular Disease	Core SUMO-Regulatory Axis	Key Mechanism	Functional Outcome	References
Cardiac remodeling and heart failure	SENP1 → JAK2/STAT3	SENP1 suppresses STAT3 phosphorylation and nuclear translocation; deficiency exacerbates pressure overload-induced hypertrophy and fibrosis.	Attenuation of pathological hypertrophy, apoptosis and cardiac deterioration	[91]
	SENP1 → SUMO1 → TEAD1 (K173)	SENP1 deSUMOylates TEAD1; a deSUMOylation-mimetic mutant aggravates hypertrophy and oxidative stress via the NRF2–HMOX1 axis.	TEAD1 SUMOylation antagonizes pathological cardiac hypertrophy	[54]
	Ang II → MEL18 → HSF2 deSUMOylation → IGF-IIR	Ang II induces MEL18-mediated HSF2 K82 deSUMOylation, activating IGF-IIR and driving hypertrophy.	Promotes pathological cardiac hypertrophy	[92]
	SENP5	SENP5 overexpression induces cardiomyocyte apoptosis and cardiomyopathy.	Mitochondrial fission, apoptosis and cardiomyopathy	[78]
	SUMO1 → SERCA2a (K480/K585)	SUMO1 stabilizes SERCA2a and enhances ATPase activity; both are reduced in failing hearts. SUMO1 gene therapy improves cardiac function in large-animal models.	Improved SR Ca^2+^ reuptake, myocardial contractility and attenuated heart-failure phenotype	[49,51,93]
	UBC9-dependent global SUMOylation	Cardiac UBC9 upregulation improves proteostasis and autophagy, reducing protein aggregation and myocardial remodeling.	Improved myocardial contractility and survival	[81,94]
Cardiac fibrosis	SENP1 → KLF4	SENP1 deSUMOylates KLF4 in aged hearts, derepressing TGF-β1/SMAD3 and promoting fibroblast senescence.	Promotes fibroblast senescence and cardiac fibrosis	[55]
	UBC9/RNF4 → PML SUMOylation	RNF4 mediates poly-SUMOylated PML degradation; modulating UBC9/RNF4 alters PML SUMOylation and fibrogenesis.	Regulation of collagen deposition; RNF4 is anti-fibrotic	[90,95]
Myocardial ischemia/reperfusion (I/R) injury	SENP1 → HIF-1α	SENP1 stabilizes HIF-1α via deSUMOylation; loss accelerates HIF-1α degradation and increases cell death.	SENP1-mediated HIF-1α stabilization confers hypoxic cardioprotection	[96]
	SENP3 → Drp1	SENP3 deSUMOylates Drp1, promoting mitochondrial fission and apoptosis; Drp1 SUMOylation is protective.	Exacerbation of mitochondrial injury; Drp1 SUMOylation protects	[38,97]
	SENP3 subcellular redistribution	Ischemia enriches SENP3 in the nucleus; knockdown increases reperfusion cell death, indicating compartment-dependent protection.	Time- and compartment-dependent cardioprotection	[98]
	SUMO2 → nuclear γ-actin	SUMO2 modification of nuclear γ-actin promotes DNA damage repair; elevated SUMO2 is cardioprotective.	Attenuation of DNA damage and myocardial injury	[99,100]
	PIAS1 → PPARγ SUMOylation	PIAS1 SUMOylates PPARγ, preserving NF-κB suppression; PIAS1 decline after I/R reduces this protection.	Reduction in I/R injury and inflammatory damage	[34]
	HDAC4 SUMOylation–ubiquitination/proteasomal degradation axis	HDAC inhibition promotes HDAC4 SUMOylation and facilitates its ubiquitination and proteasome-dependent degradation. Loss of HDAC4 is associated with reduced oxidative stress and attenuation of hypoxia/reoxygenation-induced cardiomyocyte injury.	Reduced cardiomyocyte injury and apoptosis, with improved cellular survival after hypoxia/reoxygenation stress.	[101,102]
	FXR SUMOylation	FXR SUMOylation suppresses its transcriptional activity and SHP upregulation; reduced SUMOylation during I/R aggravates injury.	FXR SUMOylation alleviates I/R injury	[103]
Arrhythmia	SENP2 → Kv7.1 (K424)	SUMOylation of Kv7.1 at Lys424 shifts the voltage dependence of IKs activation toward more depolarized potentials, with each SUMO contributing an approximately +8 mV shift; SENP2 modulates the SUMOylation state of Kv7.1.	Altered Kv7.1 SUMOylation changes IKs activation gating and may influence cardiac repolarization and arrhythmia susceptibility.	[104]
	SUMO1 → Kir2.1	Acute hypoxia activates the redox-sensitive SUMO1/UBC9 axis, rapidly SUMOylating Kir2.1 at Lys49. This modification blunts PIP2-mediated channel activation, suppressing cardiac IK1.	IK1 suppression destabilizes the resting membrane potential and final repolarization, generating a proarrhythmic substrate under acute hypoxic/ischemic stress.	[105]
	UBC9/SUMO1–3 → Kv1.5 SUMOylation → depolarized steady-state inactivation → preserved IKur function	Kv1.5 undergoes SUMOylation at two conserved cytoplasmic lysine residues (Lys221 and Lys536) by SUMO1/2/3 via UBC9. Loss of SUMOylation—by mutating conjugation sites or expressing SENP2—produces a selective ≈15-mV hyperpolarizing shift in the voltage dependence of steady-state inactivation, without altering current density or activation gating	Altered Kv1.5 inactivation gating may affect IKur function and atrial repolarization, potentially contributing to arrhythmogenic susceptibility.	[106]
	UBC9/SUMO–TRPM4 axis	The TRPM4 E7K mutation impairs channel endocytosis, resulting in increased constitutive SUMOylation and enhanced TRPM4 surface expression. Other gain-of-function TRPM4 variants have been associated with inherited cardiac conduction disease, although their specific dependence on SUMOylation remains unclear.	Increased TRPM4 cell-surface abundance and channel activity may contribute to progressive familial heart block and other cardiac conduction abnormalities.	[107,108]
Viral myocarditis	TRIM29 → SUMO1-mediated PERK SUMOylation	CVB3 infection upregulates TRIM29, which interacts with PERK and promotes its SUMOylation, thereby stabilizing PERK and enhancing PERK-mediated ER-stress signaling. SUMO1 is the predominant SUMO paralogue implicated in this regulation.	Promotes cardiomyocyte ER stress, apoptosis, and inflammatory responses; TRIM29 deficiency or PERK inhibition attenuates viral myocarditis and improves cardiac outcomes in mice.	[109]
Atherosclerosis	p90RSK → SENP2 → ERK5/p53	p90RSK phosphorylates SENP2 (T368), promoting nuclear export and reducing ERK5/p53 deSUMOylation; SENP2 loss accelerates plaque formation.	Leukocyte rolling, endothelial apoptosis and plaque progression.	[59,60]
	SENP3 → NLRP3	ox-LDL downregulates SENP3, increasing NLRP3 SUMOylation and inflammasome activation.	Loss of SENP3 increases NLRP3 SUMOylation and inflammasome activation in ox-LDL-stimulated macrophages and promotes foam-cell formation.	[57]
	ERK5 SUMOylation (K6/K22)	Disturbed flow increases ERK5 SUMOylation, which inhibits ERK5/MEF2 transcriptional activity and reduces KLF2/eNOS expression; steady laminar flow does not induce this SUMOylation.	Endothelial dysfunction, pro-inflammatory phenotype and atherosclerosis	[59,62]
	SUMOylation → TFEB	ox-LDL activates SUMO E3 ligases to SUMOylate TFEB, promoting cytoplasmic retention via 14-3-3 binding and recruiting HDAC1/2 co-repressors; this suppresses lysosomal biogenesis, autophagy and cholesterol efflux.	Promotes macrophage foam-cell formation, lipid deposition and atherosclerotic plaque progression	[58]
	Disturbed flow → FAK (K152) SUMOylation	Disturbed blood flow induces FAK K152 SUMOylation, activating a FAK–NF-κB positive-feedback loop that amplifies ROS production.	Upregulation of ICAM-1, TNF-α and endothelial inflammation, driving atherogenesis and endothelial senescence	[110]
	LXR SUMOylation transrepression	Agonist-bound and SUMOylated LXR recruits NCoR/SMRT co-repressor complexes to NF-κB at inflammatory gene promoters via protein–protein interactions, independent of DNA binding.	Transrepression of pro-inflammatory genes in macrophages, suppression of innate immune response and promotion of reverse cholesterol transport	[56]
	PIAS1 → eNOS SUMOylation	PIAS1 SUMOylates eNOS, preserving NO bioavailability; lost under palmitate or oxidative stress.	Preservation of endothelial homeostasis	[35]
	PIAS3 → VSMC phenotypic switching	PIAS3 deficiency promotes VSMC synthetic switching and plaque formation via STAT3	Vascular remodeling and plaque progression	[65]
	AMPKα2 → GPR120 SUMOylation	AMPKα2 modulates GPR120 SUMOylation, mediating fish oil’s anti-atherosclerotic effect	Suppression of vascular inflammation and plaque progression	[111]
	LRH-1 SUMOylation→ PROX1	SUMOylated LRH-1 binds PROX1, suppressing hepatic reverse cholesterol transport	Increased atherosclerosis susceptibility	[64]
	p53 SUMOylation	Disturbed flow induces PKCζ-dependent p53 SUMOylation and nuclear export, triggering endothelial apoptosis.	Endothelial apoptosis and plaque instability	[61]
Hypertension	SUMO1 → ATF3 (K42)	Ang II increases ATF3 and SUMO1 in endothelial cells and in the aortic endothelium of hypertensive mice. SUMO1-dependent modification of ATF3 at K42 reduces ATF3 ubiquitination and stabilizes ATF3, thereby enhancing inflammatory signaling and contributing to endothelial dysfunction.	Endothelial inflammation, reduced NO bioavailability, impaired vasodilation and vascular dysfunction	[112]
	SENP3 → β-catenin	Oxidative stress and Ang II increase SENP3 in vascular smooth muscle cells. SENP3 deSUMOylates β-catenin and promotes its stabilization, thereby enhancing VSMC proliferation and migration and contributing to vascular remodeling.	VSMC proliferation and migration, vascular remodeling and hypertension-associated vascular dysfunction	[113]
Transplant arteriosclerosis	SENP1 → GATA2/IκBα deSUMOylation	Inflammation induces endothelial SENP1, which deSUMOylates GATA2 and IκBα; this increases GATA2 stability/DNA-binding and NF-κB activity, synergistically upregulating adhesion molecules.	Augmented endothelial activation, leukocyte recruitment and neointima formation; endothelial SENP1 knockout attenuates graft arteriosclerosis	[63]
Diabetic cardiomyopathy	MEK → XBP1s phosphorylation-dependent SUMOylation	High glucose promotes XBP1s phosphorylation-dependent SUMOylation, blocking nuclear translocation; U0126 limits this.	Attenuation of myocardial injury, fibrosis and cardiac dysfunction	[82]
Pulmonary arterial hypertension	SENP1–FIS1 SUMOylation/deSUMOylation axis	SENP1 deSUMOylates FIS1 and preserves MFN2 oligomerization, inhibiting mitochondrial fission; lost under chronic hypoxia.	Maintenance of endothelial function; promotion of pulmonary vascular remodeling under chronic hypoxia	[114]

**Table 2:** Evidence Levels, Mechanistic Axes, and Translational Status of SUMO Research in Cardiovascular Disease.

Disease/Context	SUMO–Substrate Axis	Evidence Model	Direction of Effect	Functional Endpoint	Evidence Level	Translational Status	References
Heart failure	SUMO1–SERCA2a	Human failing hearts, mouse and large-animal models; cardiomyocytes	Protective	Improved SERCA2a activity, Ca^2+^ handling, and contractility	Strong preclinical	Most advanced cardiovascular SUMO therapeutic concept; no established cardiovascular SUMO-targeted therapy	[49,50,51]
Cardiac hypertrophy	SUMO1–SIRT1/PGC-1α; SUMOylation of TEAD1 and C/EBPβ	Cardiomyocytes, rodent models, and human cardiac samples	Context-dependent; generally protective when adaptive SUMOylation is restored	Cardiomyocyte hypertrophy, metabolic remodeling, and cardiac function	Moderate to strong preclinical	Target-validation stage; substrate-selective intervention and cardiac-specific delivery remain necessary	[52,53,54,80]
Ischemia/reperfusion injury	SENP3–Drp1; SUMOylated Drp1; MAPL–Drp1	Cultured cells and rodent myocardial I/R models	Context-dependent	Mitochondrial fission, apoptosis, infarct size, and mitochondrial integrity	Moderate preclinical	Timing and direction of SENP3/Drp1 modulation remain unresolved; intervention may be highly dependent on ischemic versus reperfusion phase	[38,39,97,98]
Ischemia/reperfusion	SUMO2–γ-actin; PIAS1–PPARγ; SENP1–HIF-1α	Cardiomyocytes and rodent models	Mostly protective	DNA-damage repair, inflammatory signaling, oxidative stress, and cardiomyocyte survival	Moderate preclinical	Multiple protective axes have been reported, but their relative importance and therapeutic window require validation in clinically relevant models	[34,96,99,100]
Atherosclerosis	ERK5/p53–SENP2	Endothelial cells, disturbed-flow models, and atherosclerotic animals	Mostly pathogenic under disturbed flow	Endothelial dysfunction, apoptosis, inflammatory activation, and plaque formation	Moderate preclinical	Cell-targeted intervention is needed because systemic SENP2 or SUMO modulation may produce opposing vascular effects	[59,60,61,62]
Atherosclerosis	SENP3–NLRP3	Macrophages, ox-LDL-treated cells, and experimental foam-cell models	Protective when SENP3 activity is restored	Foam-cell formation, NLRP3 SUMOylation, and inflammasome activation	Early preclinical	Requires validation in appropriate in vivo atherosclerosis models and human plaque material	[41,57]
Atherosclerosis	TFEB SUMOylation; AMPKα2–GPR120 SUMOylation; LRH-1/PROX1	Macrophages, hepatic, vascular, and mouse models	Predominantly pathogenic for TFEB SUMOylation; protective for GPR120 SUMOylation	Lysosomal activity, foam-cell formation, lipid metabolism, and reverse cholesterol transport	Moderate preclinical	SUMOylation effects are substrate-specific; broad pathway modulation is unlikely to be therapeutically appropriate	[58,64,111]
Pulmonary hypertension	FIS1/Drp1 SUMOylation–deSUMOylation and mitochondrial dynamics	Human samples, endothelial cells, and hypoxic animal models	Adaptive in short-term hypoxia; potentially pathogenic during persistent remodeling	Mitochondrial integrity, endothelial survival, vascular remodeling, and pulmonary pressure	Relatively strong preclinical	Duration-dependent transition remains unresolved; the axis should be described as FIS1/Drp1 SUMO regulation rather than solely SENP1–FIS1/MFN2 unless direct SENP1–MFN2 evidence is added	[114,120]
Arrhythmia and cardiac conduction disease	SUMOylation of KCNQ1/Kv7.1, Kv1.5, and Kir2.1; TRPM4 genetic channelopathy	Electrophysiological studies, heterologous expression systems, animal models, and genetic studies	Channel-specific	Ventricular repolarization, membrane excitability, conduction, and action-potential duration	Mechanistic but heterogeneous	Clinical relevance requires validation in patient-derived cardiomyocytes, genotype–phenotype studies, and prospective clinical cohorts; TRPM4 evidence is currently mainly genetic rather than SUMO-mechanistic	[104,105,106,107]
Cardiac fibrosis and remodeling	PML–SUMO/UBC9/RNF4; SUMOylation of KLF4	Cardiac fibroblasts, rodent models, and pressure-overload/aging-related models	Context-dependent	Fibroblast activation, extracellular-matrix deposition, and ventricular remodeling	Early to moderate preclinical	Fibrosis-directed therapy will likely require cell- and substrate-specific modulation rather than global SUMO inhibition or activation	[55,95]
Mitochondrial cardiomyopathy and proteotoxic stress	UBC9-mediated global SUMOylation; SENP1–MEF2C deSUMOylation	Cardiomyocytes, transgenic mice, and human failing-heart observations	UBC9-mediated SUMOylation is protective; SENP1 induction is associated with cardiomyopathy	Protein aggregation, autophagy, mitochondrial function, fibrosis, and cardiac survival	Moderate preclinical	Therapeutic translation remains constrained by the pleiotropic effects of manipulating the global SUMO machinery	[40,94]
Biomarker development	SUMO1/SUMO2/3, UBC9, SENPs, and substrate-specific SUMOylation signatures	Human tissue studies, animal models, and proteomic analyses	Disease- and substrate-dependent	Diagnosis, prognosis, treatment response, and patient stratification	Exploratory	No clinically validated cardiovascular SUMO biomarker; standardized assays, longitudinal cohorts, and prospective validation are required	[44,77,131]

## Data Availability

No new data were created or analyzed in this review. Data availability is therefore not applicable.

## Abbreviations

SUMO, small ubiquitin-like modifier; SENP, sentrin/SUMO-specific protease; E3, SUMO E3 ligase; SERCA2a, sarco/endoplasmic reticulum Ca^2+^-ATPase 2a; NF-κB, nuclear factor kappa B; NLRP3, NLR family pyrin domain containing 3.
